# Genetics and Genomics of Gastroschisis, Elucidating a Potential Genetic Etiology for the Most Common Abdominal Defect: A Systematic Review

**DOI:** 10.3390/jdb12040034

**Published:** 2024-12-19

**Authors:** John P. Marquart, Qian Nie, Tessa Gonzalez, Angie C. Jelin, Ulrich Broeckel, Amy J. Wagner, Honey V. Reddi

**Affiliations:** 1 Department of Surgery, Medical College of Wisconsin, Milwaukee, WI 53226, USA; jmarquart@mcw.edu (J.P.M.); awagner@childrenswi.org (A.J.W.); 2 Department of Pathology, Medical College of Wisconsin, Milwaukee, WI 53226, USA; qnie0207@gmail.com (Q.N.); tessagonzalez523@gmail.com (T.G.); 3 Division of Maternal Fetal Medicine, Johns Hopkins Hospital, Baltimore, MD 21287, USA; ajelin1@jhmi.edu; 4 Department of Pediatrics, Medical College of Wisconsin, Milwaukee, WI 53226, USA; ubroeckel@mcw.edu

**Keywords:** gastroschisis, genetic etiology, systematic review

## Abstract

(1) Background: The exact etiology for gastroschisis, the most common abdominal defect, is yet to be known, despite the rising prevalence of this condition. The leading theory suggests an increased familial risk, indicating a possible genetic component possibly in the context of environmental risk factors. This systematic review aims to summarize the studies focused on the identification of a potential genetic etiology for gastroschisis to elucidate the status of the field. (2) Methods: Following the PRISMA-ScR method, Pubmed and Google Scholar were searched, and eligible publications were mined for key data fields such as study aims, cohort demographics, technologies used, and outcomes in terms of genes identified. Data from 14 human studies, with varied cohort sizes from 40 to 1966 individuals for patient vs. healthy controls, respectively, were mined to delineate the technologies evaluated. (3) Results: Our results continue the theory that gastroschisis is likely caused by gene–environment interactions. The 14 studies utilized traditional methodologies that may not be adequate to identify genetic involvement in gastroschisis. (4) Conclusions: The etiology of gastroschisis continues to remain elusive. A combination of omics and epigenetic evaluation studies would help delineate a possible genetic etiology for gastroschisis.

## 1. Introduction

Gastroschisis is the most common congenital abdominal defect, typically diagnosed prenatally by the sonographic finding of the fetal intestines herniated lateral to the umbilicus. Alarmingly, the prevalence of gastroschisis is on the rise, with a recent study evaluating gastroschisis cases in the state of California during the period 1995–2012, reporting that the prevalence has increased from 1.5 per 10,000 births to 5.3 per 10,000 births during this period [1]. Despite the rising prevalence of gastroschisis, the exact etiology remains elusive with several proposed pathological theories [2].

Although, for gastroschisis, the leading theory suggests a likely disruption-type defect, multiple studies demonstrate an increased familial risk, indicating a possible genetic component [3,4,5,6] possibly in the context of environmental risk factors. There are links between the environment and epigenetics [7], suggesting that an environmental etiology could be captured with methylation studies. Over the years, a variety of genomic methodologies have been utilized to evaluate the genetic contribution to gastroschisis [7,8,9,10,11,12]. Sanger sequencing and genotyping were used to identify single nucleotide variants (SNVs) at specific genetic loci, with limited success [10]. With the development of next-generation sequencing (NGS) technology, AI-driven bioinformatics pipelines, and variant interpretation and analysis platforms, testing methodologies for rare disease have evolved in recent years [13]. The discovery of a genetic etiology requires comprehensive tools that provide an in-depth insight into the genomics of gastroschisis. Whole Exome Sequencing (WES), which provides a comprehensive investigation of the coding variants in the human genome including SNVs, is increasingly utilized for rare diseases such as neurological disorders as well as gastroschisis [11,14]. Although WES only represents 2–5% of the genome, it covers 85% of known disease-related variants. Whole genome sequencing (WGS), on the other hand, covers variants in both the coding and non-coding regulatory regions of the genome, enabling the identification of deep intronic variants of potential clinical significance [15,16].

Besides DNA sequencing, whole transcriptome sequencing (RNA Sequencing), whole-genome bisulfite sequencing (WGBS) and optical genome mapping (OGM) can also be used for rare disease diagnostics. By analyzing both coding and non-coding RNAs, RNA Sequencing provides a comprehensive view of the transcriptome and allows us to identify biomarkers and enriched pathways related to disease. WGBS can be applied to identify individually methylated targets on the genome scale to determine the role of epigenomics in genetic etiology. OGM enables the detection of structural variants not easily identified by WES or WGS. All technologies outlined have certain limitations that need to be considered when utilized. 

Studies evaluating the genetics and genomics of gastroschisis are highly varied, providing limited evidence with regards to associated genetic variation. A substantial portion of these publications investigate small cohorts and report on a multitude of genetic variants without definitive causative loci; however, the genetic etiology for gastroschisis is yet to be defined. As this body of evidence grows, along with improvements in testing technology, a comprehensive view of the field is needed. We aim to perform a systematic review of the literature to delineate the currently proposed genetic contribution to gastroschisis and provide recommendations on the next steps to achieve the same if a consensus is not clear.

## 2. Materials and Methods

The review methodology was guided by the PRISMA-ScR (Preferred Reporting Items for Systematic Reviews and Meta-Analyses extension for Scoping Reviews) and the review was not registered. Searches were conducted on three databases, OVID Medline [17], Scopus [18], and Web of Science [19] using the following search terms—Gastroschisis or abdominal wall defect, not omphalocele; includes genetics, genomics, genes, inheritance, etiology, restricted to documents in the English language, including reviews. The eligibility criteria for studies included were English language publications that focused on genetics and genomic studies for gastroschisis. Key data fields such as study aims, cohort demographics, types of technologies used, and outcomes were extracted, and the data were analyzed to address the objectives of the review. 

Genes evaluated in the study cohorts were uploaded to the KEGG pathway database [20] for pathway analysis. KEGG PATHWAY database is a bioinformatics resource for deciphering the genome through the interaction network of genes and pathways. Genes were also submitted to GENEONTOLOGY to identify biological processes enriched in gastroschisis. GENEONTOLOGY knowledgebase provides a computational representation about the functions of genes [21]. 

## 3. Results

### 3.1. Review of Literature

**Figure 1:**
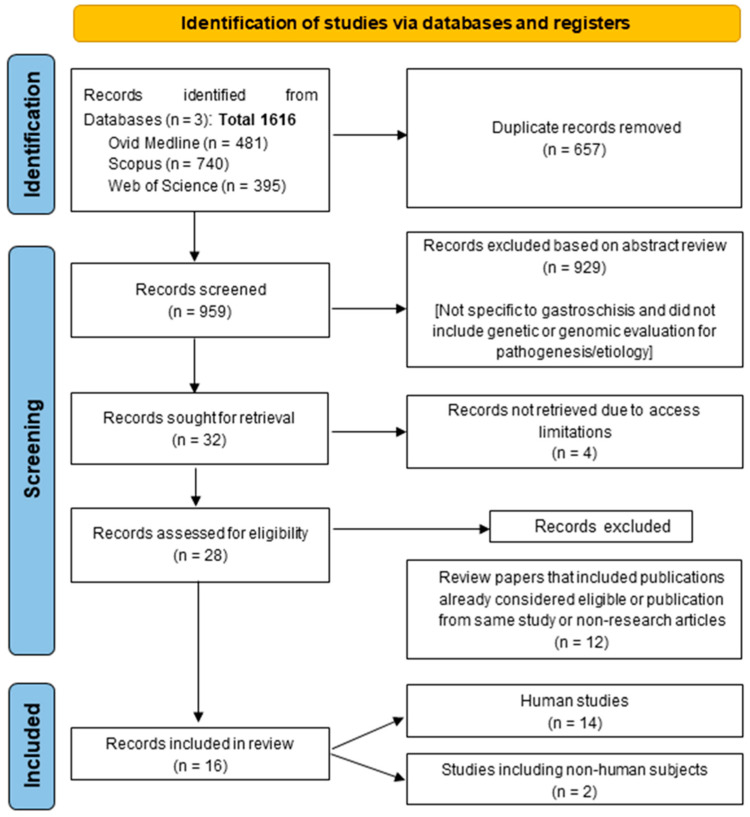
Schematic diagram outlining PRISMA-ScR model of the study screening process and number of publications included.

A total of 1616 records were identified based on the search terms described in the methods, of which 959 (59%) were shortlisted for further review after the elimination of 657 duplicate records (Figure 1). Based on the abstract and title review by JPM and QN, a further 927 (97%) records were eliminated for not pertaining to gastroschisis or not including the genetic or genomic evaluation of cohorts for the pathogenesis or etiology of gastroschisis, leaving 32 (3%) records for the final evaluation. Of the 32 records which were sought for retrieval, 4 could not be retrieved due to access limitations, resulting in 28 records being shortlisted for a final review (Figure 1). Upon a detailed evaluation, 12 of the 28 records (43%) were excluded from the study because they were review articles that included publications already considered eligible or publications from the same study or non-research articles not gastroschisis-related, bringing the final number of records included in this study to 16 (Figure 1). Fourteen of the sixteen records included human subjects, while two focused on animal models for gastroschisis. Of the fourteen human studies, ten were cohort studies, of which seven included matched controls, with varied cohort sizes from 40 to 1966 individuals for patient vs. healthy controls, respectively (Table 1). 

### 3.2. Technologies Used in Studies Reviewed

The fourteen human subjected studies were evaluated to delineate the technologies applied in gastroschisis studies (Table 1). The respective study confirmed the phenotypic overlap between Moebius syndrome and Oromandibular Limb Hypogenesis syndrome (OLHS) and widens the spectrum of associated malformations (including gastroschisis). The patients evaluated in this study were evaluated by Karyotyping and array-based comparative genomic hybridization, with the results being normal [9]. Two studies performed karyotyping to find gastroschisis-related variants at the chromosomal level [29,31]. Seven studies genotyped gastroschisis patients and their matched controls to identify variants that may be gastroschisis-related, of which six studies primarily used a polymerase chain reaction (PCR) to examine variants in specific genes [22,24,26,27,30]. In addition to PCR, these studies also applied array comparative genomic hybridization (aCGH) to screen the patient’s genome [30], a TaqMan Genotyping assay [26], multi-locus allele-specific hybridization assay [23], SNP genotyping with the HaploView program to analyze the data [25], and the Illumina OmniExpress genotyping array with >700,000 SNPs to perform the shared genomic segment analysis [9]. Additional methodologies included the use of WES on two affected half-sisters to identify novel variants that could contribute to gastroschisis [13], followed by functional bioinformatics analysis based on an SVS-PhoRand and Ensembl-Variant Effect predictor [9]. No studies utilizing RNAseq as a sole methodology were identified. 

### 3.3. Genes and Pathways Evaluated for Association with Gastroschisis

Across the 14 studies evaluated for inclusion in this review (Table 1), it was observed that study cohorts focused on specific genes (Table 1) such as *MTHFR* [8], *BMP1* [22], *AEBP1* [24], and *ICAM1* [27]. In one study that focused on variants in *MTHFR*, Factor V and prothrombin in gastroschisis patients did not find any significant role of these variants in the development of gastroschisis [8]. In contrast, another study evaluating a gastroschisis patient of Indonesian ethnicity proposed that *MTHFR* c.677C>T reduced the MTHFR enzyme activity, contributing to gastroschisis [26]. These two studies focused on different ethnicity groups, which could explain the alternate findings. Another study in Indonesia analyzed the frequency of the *ICAM1* common variant K469E in gastroschisis patients but was unable to demonstrate that *ICAM1* K469E is a genetic risk for gastroschisis [27]. On the other hand, two California population-based case-control studies suggested that there were specific *ICAM1* variants (*ICAM1* gly241arg and *ICAM1* rs281432) associated with an increased risk for gastroschisis [23,25]. Besides *ICAM1*, the study from the Torfs group [23] also identified a heterozygous/homozygous variant of *NOS3*, *NPPA*, *ADD1*, *SERPINE1*, and *SELE*, genes involved with cell–cell interactions, inflammation, or blood pressure, associated with gastroschisis. Padula [25] investigated 75 genetic variants in 20 genes and found 11 gastroschisis-associated variants in *NOS3*, *ADD1*, *GNB3*, *ICAM1*, *ICAM4*, *ICAM5*, and *NAT1* genes. 

Four studies evaluated for this review investigated the genetic cause of gastroschisis at the chromosome level using traditional karyotyping; however, no chromosomal abnormalities were observed across the 36 gastroschisis patients evaluated [29]. A copy number variant study evaluated the karyotype and microarray data of 4340 individuals that had either chorionic villous sampling or amniocentesis and identified one patient with an ultrasound anomaly in the abdominal wall. Of the 1082 fetuses with anomalies detected on the ultrasound scan, 752 had a normal karyotype. Only 1 patient had copy number variants (CNVs) notable in the ‘abdominal wall defect’ group of 40 patients. However, there was no significant correlation between this abnormality and the observed CNV [31]. Two independent case reports each evaluated a gastroschisis patient with an additionally affected organ system. In the first case, aCGH identified a 531 kb duplication of the *CAMK1D* gene in a patient with both gastroschisis and a neural tube defect [30]; however, the finding could not be confirmed by other methodologies or in prospective studies, leaving it to be unvalidated in terms of association with gastroschisis. A second clinical report documented a gastroschisis patient with pulmonary hypoplasia and Moebius syndrome evaluated by karyotyping and aCGH that identified a gene in region 13q12.2-q13 associated with Moebius syndrome [28]. No evidence of any gene associated with gastroschisis was described. A WES study of two affected half-sisters with gastroschisis and the mother and father of the proband identified 429 genes involving DNA variants co-segregating with gastroschisis [13]. Moreover, 9 of the 429 genes were predicted as high-impact in both cases, *SPATA17*, *PDE4DIP*, *CFAP65*, *ALPP*, *ZNF717*, *OR4C3*, *MAP2K3*, *TLR8*, and *UBE2NL,* in a follow-up study using expanded analysis and bioinformatic applications [13]. 

Mouse models that showed gastroschisis-like phenotypes spurred interest in genes such as *BMP1* and *AEBP1.* In 2001, a *BMP1* gene null deletion mouse model [8] showed a gastroschisis-like condition; however, a retrospective study was unable to identify any mutations on the *BMP1* gene in a cohort of 11 gastroschisis patients [22]. Similarly, knocking out the mouse *Aclp*, a different isoform of the *AEBP1* gene, resulted in a ventral wall defect similar to gastroschisis in humans. Therefore, the Feldkamp group screened *AEBP1* variants in 40 gastroschisis cases [3]. Although no significant differences were found in the frequency of the *AEBP1* variant in the gastroschisis vs. control groups, this study showed that the *AEBP1* might interact with other immune response pathway genes. This seemed to correlate with findings from another study conducted by the same group [9]. Applying the Illumina OmniExpress genotyping array with over 700,000 SNPs, the genome-wide high-density SNP data from 40 affected patients were investigated. This study identified 107 genes that have SNPs shared by the patients, 33 (32%) of which are immune pathway genes.

In addition to the above-mentioned studies, two additional studies evaluating animal models for gastroschisis were evaluated, it is important to note that these studies were not followed up with human studies to confirm findings. In a study wherein gastroschisis was surgically created in a fetal rabbit, cellular lactase expression *CRBPII (RBP2)* was quantified and found to be decreased in the intestine of affected rabbits compared to control animals [32]. A mouse study suggested that the presence of shorter telomeres associated with p53-deficiency could contribute to gastroschisis [33]. 

Overall, Table 2 summarizes the 25 genes that were evaluated using the KEGG pathway and the associated results. Not all genes were assigned with pathway information using the analysis tool. The submission of the 25 genes to GENEONTOLOGY also did not produce significant results; however, in looking at the GO biological process, the GO molecular function analysis showed that calmodulin binding is significantly enriched in this gene list with the *p* value at 6.71 × 10^−6^. 

## 4. Discussion

The diagnosis of gastroschisis is most commonly made during a prenatal ultrasound evaluation [34]. Understanding the pathogenesis and etiology that results in gastroschisis could allow for early detection and possible prevention. The results of this systematic review continue the theory that gastroschisis is likely caused by gene–environment interactions. While a monogenetic etiology has yet to be identified, this study provides insight into the various genes proposed to be involved in the etiology of gastroschisis, with *ICAM1* being the most common one to be identified (Table 1), pressing the need for targeted studies that include genomic and epigenomic evaluations in the context of environmental exposure.

A variety of environmental risk factors have been associated with the development of gastroschisis including substance abuse, dietary intake, medications, and chemical exposure [35,36,37,38,39]. Multiple studies have shown maternal smoking, drug use, and alcohol consumption to increase the risk of gastroschisis, including a recent meta-analysis [35,40,41]. Regarding the impact of a specific dietary intake on gastroschisis, a small number of studies showed an association with a higher fat intake or diminished nutrients such as alpha-carotene, glutathione, or high nitrosamines [37,42]. There have been selected animal studies focused on the impact of medications or radiation on genetically susceptible mice that may induce a multitude of defects including gastroschisis [43,44]. Overall, there has been mixed evidence for occupational chemical exposure affecting gastroschisis [45,46]. Despite this, the continued increased incidence of gastroschisis and the use of pesticides in modern farming techniques have prompted further investigation into chemical exposure as a possible risk factor for the development of abdominal defects [47,48,49,50]. Multiple studies in a variety of geographic locations have found connections between numerous chemical compounds, such as nitrate and atrazine, with an increased risk for abdominal wall defects [39,49,51,52,53,54]. Many of these studies focused on comparing surface water pesticide concentrations with birth records as well as the timing of conception due to seasonal variation, which has limitations in proving direct causation [55].

To connect environmental exposure with genetic susceptibility, there have been limited studies of the epigenetic component of gastroschisis showing variation between family members [7], suggesting that epigenetics may play a role in the etiology of gastroschisis. Applying technologies such as MethylSeq and WGBS could identify disease-causing epigenetic changes that are located on the transcription factors or enhancers [56]. Modifications of DNA such as acetylation, impacting histone regulation and thereby biological functions, could have a significant effect on pathogenicity. ChIP-Seq and ATAC-Seq can be used to investigate the alterations in chromatin configuration [57]. Additionally, gene expression differs from tissue to tissue. Whole blood, which is a preferred specimen type in most testing types, may not represent the same gene expression level in the tissues involved in gastroschisis [58]. RNA-Seq that can quantify the expression difference of genes in different conditions is an appropriate approach to support findings from the DNA-Seq. Single-cell RNA-Seq (scRNA-Seq) that detects the transcript expression in specific populations of cells can suit the purpose better [59]. However, the challenge remains in the clinical sample collection, cost, and analysis efforts.

The 14 studies evaluated for this review utilized traditional methodologies that may not be adequate to identify the genetic association for gastroschisis. With the advances in genomics, using the latest omics technologies including WGS, WGBS, and RNA-Seq could help delineate the genetic and/or epigenetic changes involved in the causation of gastroschisis. Rare disease research studying the genetic causes showed that only less than half of the studied cases can be explained by the variants on the coding region, which indicates the importance of investigating genetic alterations outside of coding regions [60]. WGS allows for the expansion of the detection range to include non-coding regulatory and splicing-relevant regions, covering variants beyond the exome [61]. In addition, CNVs, which were previously captured by array, can also be accurately detected by WGS, improving the diagnostic yield of genetic testing for rare-disease patients, identifying novel genetic alterations [62].

We acknowledge that there are limitations to this paper. The first is the inherent limitations in the study design. The studies identified in the meta-analysis associate genetic etiologies with gastroschisis and potentially lack causation. These papers could include monogenetic causes as well as potential loci of susceptibility, such as is the case of p53, which requires irradiation to invoke a phenotype. The results are not inclusive of all causes of gastroschisis as the methods of each paper differ. Possible etiologies of gastroschisis could also be missed by the chosen search criteria. 

## 5. Conclusions

Despite a variety of studies evaluating the genetic component of gastroschisis, its etiology continues to remain elusive. We did not identify reportable causative chromosomal aberrations on the karyotype or microarray, and it appears that single-gene variants rarely cause gastroschisis. Several genes have, however, been proposed to contribute either as the loci of susceptibility or as part of other complex gene–environment interactions. A combination of omics and epigenetic evaluation studies should be performed to identify the possible genetic etiology for gastroschisis.

## Figures and Tables

**Table 1:** Summary of Records Evaluated.

Study	Number of Gastroschisis Patients	Number of Controls	Technology/Methodology	Gene/Pathway
Human Studies				
Komuro, Mori [22]	11	0	PCR	*BMP-1*
Torfs, Christianson [23]	57	506	Multi-locus allele-specific hybridization assay	*ICAM1*, *NOS3*, *NPPA*, *ADD1*, *SERPINE1*, *SELE*
Feldkamp, Bowles [24]	40	0	PCR	*AEBP1*
Padula, Yang [25]	63	106	HaploView program	*NOS3*, *ADD1*, *GNB3*, *ICAM1*, *ICAM4*, *ICAM5*, *NAT1*
Makhmudi, Sadewa [26]	46	89	PCR-RFLP and TaqMan Genotyping Assays	*MTHFR*
Makhmudi, Aryandono [27]	48	88	PCR	*ICAM1*
Feldkamp, Krikov [9]	40	168	Illumina OmniExpress genotyping array	immune pathway genes
Salinas-Torres, Gallardo-Blanco [11]	2	2	WES	429 genes
Salinas-Torres, Gallardo-Blanco [13]	2	2	WES	*SPATA17*, *PDE4DIP*, *CFAP65*, *ALPP*, *ZNF717*, *OR4C3*, *MAP2K3*, *TLR8*, *UBE2NL*
Brockmann, Backes [28]	1	0	Observation	a gene located in region 13q12.2-q13
Cardonick, Broth [8]	55	182	PCR	*MTHFR*
Chabra and Hall [29]	36	0	Karyotyping	NA
Chen, Shen [30]	1	0	CGH-array and long-range PCR	*CAMK1D*
Donnelly, Platt [31]	752	1966	karyotype and chromosomal microarray	NA
Animal Model Studies				
Srinathan, Langer [32]	NA	NA	Realtime PCR	*CRBPII*
Bekaert, Derradji [33]	NA	NA	FISH	*P53*

PCR—Polymerase chain reaction, RFLP—restriction fragment length polymorphism, WES—whole exome sequencing, FISH—fluorescent in situ hybridization.

**Table 2:** Gene and Pathway Summary.

Gene Symbol	Gene Name	KEGG ID	Pathway	Effect
*ADD1*	DNA damage inducible transcript 3	K04452	hsa04010_MAPK signaling pathway	YES
*AEBP1*	AE Binding Protein 1	K21392	NA	NO
*ALPP*	Alkaline Phosphatase, Placental	NA	NA	YES
*BMP1*	growth differentiation factor 11	K22679	hsa04060_Cytokine–cytokine receptor interaction	NO
*CAMK1D*	calcium/calmodulin-dependent protein kinase I	K08794	hsa04020_Calcium signaling pathway	YES
*CFAP65*	cilia- and flagella-associated protein 65	K24226	NA	YES
*RBP2*	RNA binding protein fox-1	K14946	NA	
*GNB3*	G protein subunit beta 3	K07825	hsa04014_Ras signaling pathway	YES
*ICAM1*	intercellular adhesion molecule 1	K06490	hsa04064_NF-kappa B signaling pathway	CONFLICT
*ICAM4*	intercellular adhesion molecule 4	K06581	NA	YES
*ICAM5*	intercellular adhesion molecule 5	K06769	NA	YES
*MAP2K3*	mitogen-activated protein kinase 3	K04432	hsa04010_MAPK signaling pathway	YES
*MTHFR*	methylenetetrahydrofolate reductase	K25004	hsa01100_Metabolic pathways	CONFLICT
*NAT1*	N-acetyltransferase 1	K00622	hsa00232_Caffeine metabolism	YES
*NOS3*	coiled-coil–helix–coiled-coil–helix domain containing 3	K17563	NA	YES
*NPPA*	natriuretic peptide A	K12334	hsa04022_cGMP-PKG signaling pathway	YES
*OR4C3*	olfactory receptor family 4 subfamily C member 3	K04257	hsa04740_Olfactory transduction	YES
*TP53*	Tumor Protein P53	NA	NA	Unclear
*PDE4DIP*	phosphodiesterase 4D interacting protein	K16549	NA	YES
*SELE*	selenoprotein P	K25753	NA	YES
*SERPINE1*	plasminogen activator inhibitor 1	K03982	hsa04066_HIF-1 signaling pathway	YES
*SPATA17*	spermatogenesis-associated protein 17	K25546	NA	YES
*TLR8*	toll-like receptor 8	K10170	hsa04613_Neutrophil extracellular trap formation	YES
*UBE2NL*	ubiquitin conjugating enzyme E2 N like	K10580	hsa04120_Ubiquitin-mediated proteolysis	YES
*ZNF717*	zinc finger protein 717	K09228	hsa05168_Herpes simplex virus 1 infection	YES

## Data Availability

The original contributions presented in the study are included in the article; further inquiries can be directed to the corresponding author.
